# Impact of comorbidities on HIV medication persistence: a retrospective database study using US claims data

**DOI:** 10.7448/IAS.15.6.18063

**Published:** 2012-11-11

**Authors:** E Maiese, M Malmenäs, M Atkinson

**Affiliations:** 1 Merck Sharp and Dohme Corp, Whitehouse Station, NJ, USA; 2 Heron Evidence Development Ltd., HEMU, Stockholm, Sweden; 3 University of California, San Diego School of Medicine, San Diego, CA, USA

## Abstract

Medication persistence (MP) is important in HIV management as lifelong HIV therapy is needed and discontinuation of HIV therapy could represent a permanent loss of therapeutic options. Many factors have been shown to decrease HIV treatment persistence; however, the evidence for comorbidities has been conflicting [1,2]. This study was conducted to further explore the impact of comorbidities on HIV MP. Data from the IMS PharMetrics claims database was used. To be included, patients had to 1) be 18 years of age or older; 2) have a diagnosis code for HIV during the study period (Jan 2006–Sep 2011); 3) have a claim for at least one HIV medication during the index period (Jan 2007–Sep 2010); and 4) have continuous enrollment 12 months before and after the index date. Patients could not have a diagnosis code for pregnancy during the study period or a claim for an HIV medication during the 12 months prior to the index date. The index date was the date of the first claim of an HIV medication during the index period and all HIV medications recorded on the index date were included as the HIV index regimen. MP was defined as time to discontinuation of the HIV index therapy using a 90-day grace period. Variables statistically significant (p<0.05) in bivariate testing were included in a Cox proportional hazard model to adjust for confounding. Gender, index year, insurance provider type, number of HIV pills/day, and number of comorbidities were included in the final Cox model. A total of 3,057 patients were included in the analysis. The mean age was 43.9 yrs and 76.3% were male. The average MP was 315 days (min 92–max 365). In the Cox model, patients with 1, 2 and≥3 comorbidities had a 6% (p=0.528), 28% (p=0.014) and 31% (p=0.002), respectively, higher risk of discontinuing HIV index regimens than patients with no comorbidities. Additionally, females had a 29% (p<0.001) higher risk of discontinuing HIV index regimens than males. The analysis supports prior evidence that comorbidities decrease HIV MP. This observation may be the result of patients switching HIV medications due to drug-drug interactions from polypharmacy for managing HIV and comorbidities or due to HIV medication adverse effects. Further research should address the impact of specific HIV regimens on HIV MP among patients with comorbidities and potential differences between genders.
